# Evaluation of Forensic DNA Traces When Propositions of Interest Relate to Activities: Analysis and Discussion of Recurrent Concerns

**DOI:** 10.3389/fgene.2016.00215

**Published:** 2016-12-12

**Authors:** Alex Biedermann, Christophe Champod, Graham Jackson, Peter Gill, Duncan Taylor, John Butler, Niels Morling, Tacha Hicks, Joelle Vuille, Franco Taroni

**Affiliations:** ^1^Faculty of Law, Criminal Justice and Public Administration, School of Criminal Justice, University of LausanneLausanne, Switzerland; ^2^School of Science, Engineering and Technology, Abertay UniversityDundee, Scotland; ^3^Norwegian Institute of Public HealthOslo, Norway; ^4^Department of Forensic Medicine, University of OsloOslo, Norway; ^5^Forensic Science South AustraliaAdelaide, SA, Australia; ^6^School of Biological Sciences, Flinders UniversityAdelaide, SA, Australia; ^7^National Institute of Standards and TechnologyGaithersburg, MD, USA; ^8^Section of Forensic Genetics, Department of Forensic Medicine, Faculty of Health and Medical Sciences, University of CopenhagenCopenhagen, Denmark; ^9^Faculty of Law, University of NeuchâtelNeuchâtel, Switzerland

**Keywords:** interpretation, probative value, hierarchy of propositions, probability assignment

## Abstract

When forensic scientists evaluate and report on the probative strength of single DNA traces, they commonly rely on only one number, expressing the rarity of the DNA profile in the population of interest. This is so because the focus is on propositions regarding the source of the recovered trace material, such as “the person of interest is the source of the crime stain.” In particular, when the alternative proposition is “an unknown person is the source of the crime stain,” one is directed to think about the rarity of the profile. However, in the era of DNA profiling technology capable of producing results from small quantities of trace material (i.e., non-visible staining) that is subject to easy and ubiquitous modes of transfer, the issue of source is becoming less central, to the point that it is often not contested. There is now a shift from the question “whose DNA is this?” to the question “how did it get there?” As a consequence, recipients of expert information are now very much in need of assistance with the evaluation of the meaning and probative strength of DNA profiling results when the competing propositions of interest refer to different activities. This need is widely demonstrated in day-to-day forensic practice and is also voiced in specialized literature. Yet many forensic scientists remain reluctant to assess their results given propositions that relate to different activities. Some scientists consider evaluations beyond the issue of source as being overly speculative, because of the lack of relevant data and knowledge regarding phenomena and mechanisms of transfer, persistence and background of DNA. Similarly, encouragements to deal with these activity issues, expressed in a recently released European guideline on evaluative reporting (Willis et al., 2015), which highlights the need for rethinking current practice, are sometimes viewed skeptically or are not considered feasible. In this discussion paper, we select and discuss recurrent skeptical views brought to our attention, as well as some of the alternative solutions that have been suggested. We will argue that the way forward is to address now, rather than later, the challenges associated with the evaluation of DNA results (from small quantities of trace material) in light of different activities to prevent them being misrepresented in court.

## 1. Introduction

### 1.1. Topic of the discussion

This paper deals with perceived obstacles and potential solutions in the evaluation of the probative value of forensic biology results, such as DNA profiles^1^, when the competing propositions of interest relate to activities rather than the source of the recovered trace material. So-called source level propositions deal with the origin of traces, for example, “The bloodstain on the broken window comes from Mr. A” vs. “The bloodstain comes from an unknown person^2^.” In turn, examples of so-called activity level propositions, as they are understood here, are “Mr. A punched the victim” vs. “The person who punched the victim shook hands with Mr. A,” or “Mr. A had sex with Ms. B” vs. “Mr. A and Ms. B attended the same party, and they had social interaction (i.e., shook hands) only^3^.” At first sight, the evaluation of DNA results given (sub-) source level propositions is often more straightforward because it requires little more than a careful assessment of the rarity of the corresponding analytical features in the relevant population, and because well accepted models, data and software are available. This is different in the context of activities, as can be shown through formal analyses of expressions for probative strength (e.g., Evett, 1984; Evett et al., 2002). These formulaic developments show that it is necessary to extend the consideration to additional aspects, such as background presence of DNA and phenomena of transfer and persistence. Such additional factors are widely regarded as challenging and difficult to overcome (see Meakin and Jamieson, 2013 for a review). In essence, the concern perceived among practitioners is that the additional factors cannot be assessed appropriately (e.g., because of a lack of data). Therefore, the evaluation of DNA profiling results with respect to propositions regarding activities is considered not feasible or robust enough, and should be advised against. Clearly, following precepts and ethical considerations stipulated by codes of conduct (e.g., ENFSI Board, 2005; National Commission on Forensic Science, 2016), scientists are driven by their intention to inform recipients of expert information to the best of their knowledge so that no unwarranted conclusions will be reached. Laudable though this aim might be, there remains considerable diversity in opinions about the extent to which such results may be used, and how to report them.

Evaluation of scientific results with activity level propositions represents an important topic for current forensic science practice. Rather than dismissing the topic, we believe that it is necessary for the field as a whole to engage actively and submit the underlying issues to detailed analyses. The discussion presented in this paper aims at promoting and facilitating mutual understanding, which we hope will enable progress along new and feasible avenues. Not pursuing this topic bears the risk of leaving recipients of expert information without guidance. Reliance on recipients' own devices is prone to conclusions that are based on (sub-) source level propositions being wrongly carried over to conclusions about activity level propositions.

### 1.2. Objectives

The aim of this paper is twofold—firstly, to discuss recurrent concerns and reservations about, and sometimes fear of, evaluations of probative value with respect to propositions about activities and, secondly, to discuss alternative “solutions” that have been offered. Although we do not contest that challenges can arise in practice, we will argue that the central claims of the critiques cannot be sustained across the broad diversity of aspects of interpretation with activity level propositions. In particular, we will argue that some of the perceived drawbacks are sometimes the result of misunderstanding about the role of forensic scientists and forensic science in the legal process. Further, we will stress that it does not follow from the perceived deficits that evaluations given activity level propositions should be abandoned altogether, but areas need to be defined where additional research and support for practitioners is needed. The main motivation for this perspective is that it is by helping address activity level propositions that forensic science can offer more value to the criminal justice process, in terms of more focused and useful contributions. Moreover, this is a good way to assess all the scientific results^4^ in any one case, ensuring that conclusions given by scientists do not run the risk of misleading at the evaluative stage^5^. The suggested framework provides a transparent way for experts, whether they be appointed by the court or hired by the prosecution or defense, to evaluate a case, where differences of opinion may be discussed and resolved. Courts need to provide a forum for such discussions to take place.

The paper is organized as follows. In Section 2 we present and discuss several recurrently expressed concerns. We broadly group these discussion points in three subsections, dealing with propositions (Section 2.1), data (Section 2.2) and aspects of reporting (Section 2.3). These three themes often arise in a hierarchy. Indeed, without propositions, it will be difficult for scientists to know how forensic science might help in a case. Next, with no or limited data, scientists may be reluctant to evaluate their findings. Finally, scientists may disagree about the form and content of scientific reporting, i.e., what exactly—if anything—should be reported. The issues and possible solutions explored in the three subsections are intimately linked and cannot truly be considered in isolation. Inevitably, there is some repetition between the subsections. Conclusions are presented in Section 3.

## 2. Discussion of selected issues

### 2.1. Propositions: “we don't know what the exact activities are”^6^

It is often the case that scientists will be informed about the competing propositions regarding activities alleged by the parties only at trial, if at all (Risinger, 2013). It is generally understood that the propositions of interest are “(…) set by the specific case circumstances or as indicated by the mandating authority” (Willis et al., 2015, p. 6), but limited cooperation by the defense often represents an obstacle in practice^7^. If propositions are not available, or only one proposition is available, scientists should make every effort to obtain relevant information^8^ regarding the position of each party involved in the process (see Willis et al., 2015 for complete guidance on how to deal with the absence of propositions), because without at least a pair of propositions, it is impossible to evaluate forensic observations in a balanced way.

It is a common misconception that the scientist who is evaluating the observations in light of competing posited activities needs to know every aspect of what has allegedly happened. For example, if it is alleged that the suspect grabbed the victim, aspects of DNA transfer will be important in considering activities. Only rarely will the scientist be provided with an exact recount of the position of grabbing, the force used to grab, the exact length of time the struggle lasted, and so on, to cover all aspects of the alleged encounter. However, there are several aspects that should be considered. Firstly, the manner in which the activities are, or have been, set up in controlled experiments to mimic the activity of interest are likely also to have similar aspects of uncertainty. Therefore, the uncertainty arising from many of the unmeasurable (and unknowable) aspects of the alleged activities will present themselves in the spread of the obtained data. Secondly, controlled experiments can be set up to study the impact that different factors have on the transfer of DNA during the activity in question. It may be that there is a large enough amount of variation in one aspect of the activity, such as the shedder status of the individual, that all others (such as the length or vigor of contact or when the person of interest last showered, etc.) have a negligible effect on the evaluations. In that case, this level of activity resolution is not required. Thirdly, if a number of aspects are found to have a considerable impact, then they can be included in the logical framework used to evaluate the findings. If the actual states of these important factors are not known (or not provided) by either party then they can be incorporated by considering all possible states within the evaluation, weighted by probabilities informed either by data from controlled experiments, supplemented by the analysts' knowledge, which should be available for disclosure and auditing (see also Section 2.2.2). If further information is provided later on, then the evaluation can be updated accordingly. Alternatively, sensitivity analyses can be used to determine how much of an effect any one of the unknown factors of the activities has on the value of the findings (Biedermann and Taroni, 2006). If the strength of the observations is particularly sensitive to some aspects then efforts should be made to find additional information about those aspects rather than every aspect of the activity. If scientists do not have such specialized scientific knowledge, the court will be even less likely to have such knowledge.

### 2.2. Data

#### 2.2.1. “Because each case has its own features, the use of numerical values from experimental studies performed under controlled (laboratory) conditions cannot be used for evaluation in real-life cases”

This is a general claim (e.g., Jamieson, 2011) that conflicts with scientific practice. Throughout science, experiments are conducted in trials that reflect not all, but the essential, features of the problem at hand. Clearly, medical treatments administered to patients have not previously been “tested” on those particular individuals, but on other patients with the same disease. Similarly, the safety of consumer products (e.g., cars) is carefully assessed not by end-users but prior to marketing in a range of situations reflecting end-user profiles. Turning to forensic science, such as glass analysis, the phenomenon of transfer has been studied for a variety of factors, such as the mode of breaking (e.g., the number of blows), window dimension, etc. to build a model usable for assigning transfer probabilities in cases with features covered by this model (Curran et al., 1998, 2000). In the context of DNA, studies have been conducted to examine the rates of transfer, for example between shooters and guns (Polley et al., 2006), but also in more general situations (e.g., Phipps and Petricevic, 2007; Daly et al., 2012; Jones et al., 2016; Samie et al., 2016). So, when a scientist is faced with assigning a probability for finding trace material given the proposition of handling an object by a person of interest (e.g., the *activity* of discharging a firearm), we do see no harm in referring to studies that have focused on rates of transfer not exactly the same in the alleged circumstances of the case. Although some features of the individual case at hand may differ, nothing will prevent the scientist from also judging that some additional case-tailored experiments should be conducted in order to extend their knowledge and understanding, but case backlogs and limited resources may render this difficult. Besides, if a scientist refuses to assign a probability of observing some finding under a given set of propositions, there is a risk that the fact-finder will nonetheless assign such probabilities according to their own unaided judgment which, as highly-publicized past cases suggest, will often play out to the detriment of the defendant.

Contrary to a widely held view (e.g., Budowle et al., 2012), the availability of “hard” (i.e., numerical) data is not a necessary requirement for probability assignment. That is, the absence of data does not mean that no probability can be assigned. How is this possible? To understand this, it is important to recall that scientists can also derive probability assignments from their understanding of the principles of the process at hand, formulated in terms of a model. For example, weather forecasters cannot “play” the next day over and over again to find the number of times there will be rain on exactly the next day. This makes no sense, essentially because there is only one tomorrow and its weather will be observable only once tomorrow arrives. And yet, scientists are able to formulate previsions about the state of the atmosphere based on related data (e.g., today's weather) and their understanding of the relevant science and technology. Similarly, forensic scientists can make probability statements for outcomes based on their general understanding of a phenomenon. In genetics, for instance, there is knowledge about population structure and the ways in which genetic traits are passed on between generations. Take, for example, a crime stain with a haplotype for which no other occurrence is found in a relevant database: clearly, the observed relative frequency (i.e., zero) in the database does not mean that we should retain a zero probability for observing the same haplotype in another person from the population of interest. Instead, a value for the haplotype population proportion can be obtained using reasonable assumptions (Brenner, 2010).

In reply to the above, it may be suggested that forensic genetics is not an insightful example because of the sophisticated mathematical models available in this area, so let us consider the case of DNA transfer phenomena. Here, many studies have found, for example, that the quantity of DNA recovered after touching (i.e., primary transfer) a surface with bare hands varies approximately between zero and more than 150 ng, depending on the experimental conditions (e.g., Daly et al., 2012). But can such knowledge help formulate probabilities of finding DNA under the assumption of secondary transfer? We contend that it can, by constructing an argument. It is known, for example, that secondary transfer is conditioned on the amount of DNA transferred initially. Hence, when a quantity above, say, 200 ng is found—something not typically expected when touching with bare hands (i.e., primary transfer)—it can be argued that this should be considered an even less probable event assuming secondary transfer. Thus, despite the fact that explicit data for a particular secondary transfer scenario may not be available, forensic scientists can still convey domain-relevant knowledge intelligibly in probabilistic terms. It is important, however, to ensure that the data are relevant to the analytical methods used in the case of interest, because detected quantities after secondary transfer will be sensitive to the method used to collect material and to detect DNA. It will also be necessary to ensure that probabilistic models, such as Bayesian networks (see also below), used to interpret findings, are informed by such relevant data and available for auditing.

There remain, however, justified questions as to where and how to obtain data. Currently, results from empirical research are mainly published in peer reviewed scientific journals, but there is no systematically organized body of research. The idea of developing a knowledge base (Evett, 2015), to be shared among scientists who all contribute to the system, would thus represent a major contribution to strengthen the data-supported evaluations.

In summary, probability assignment is feasible and justifiable even with limited data, but must be amenable to a critical analysis. Further, despite the fact that data are collected under conditions that do not exactly reflect all the features of the case at hand, this does not preclude, in principle, these data from being used at least to some extent^9^. Of course, this does not mean that any data are acceptable to support any claim, but—as noted above—data that the scientist regards pertinent. Moreover, expert assessment is not exclusively given by data alone; in fact, it never is because, while reference data have been collected in controlled studies, the probabilities we assign relate to one-off individual incidents. Instead, scientists use data to inform their judgment, by constructing an argument, explaining what data they have used, to what extent and why.

A topic related to the above viewpoint is the question of how to conduct assessments, i.e., reasoning in the face of uncertainty, whatever the data may be and the extent to which they are available. Often, one can note that scientists shy away from seeking support from conceptual devices that could help them structure their reasoning and, thus, avoid the impression of being overwhelmed by the inferential complexity of the evaluative task. It is thus worthy to mention one common method, known as Bayesian networks (BNs) (Evett et al., 2002; Biedermann and Taroni, 2012; Fenton and Neil, 2013; Taroni et al., 2014), for pulling together many aspects of information that need to be considered when activity level propositions are of interest. BNs are a graphical tool in which the problem can be constructed in a framework of logical inference^10^. The formulation of such a framework does not rely on having any data, it will in fact inform the analyst of what data is required in the evaluation of the findings. The formulation of a framework of inference should be the first step in any evaluation given activity level propositions. Sometimes, however, analysts claim that an insufficient amount of data exists, and they do so even before they know what data is actually required to help address the issues that are of interest in the case. Also, once constructed, and lack of data is found to be an issue for some aspects of the evaluation, there are still several avenues open to the scientists. These include the weighing of the various states that potential factors may take in terms of probabilities, informed by the scientist's documented knowledge and experience, and then conducting sensitivity analyses to determine how the evaluation changes as those probabilities vary over plausible ranges.

Reluctance to such introspective thinking, and expert probability elicitation in general, is surprising and odd to see among those scientists who would find no objection to be asked and to give opinions in court about the probabilities of various competing activities, regardless of whether relevant data exists, and regardless of whether they have undertaken some activity level consideration. In this situation, analysts are likely to express themselves non-numerically in the form of an answer such as “that is improbable,” “I don't believe that is likely to have happened,” “I think that sort of transfer is barely feasible,” etc. So, analysts who would be willing to express themselves in such probabilistic terms about propositions, but refuse to provide probabilities for findings given propositions, exhibit an inherently contradictory position. We thus maintain that analysts who have considered their findings in a framework of logical inference, using experimentally derived data to assign probabilities and varying assignments for influencing factors will be in a much better position to usefully inform the court. This will include any limitations that characterize the data actually used (as well as detailing the information available to the scientist at the time of writing the report, see also Section 2.3.3).

#### 2.2.2. “Expert professional experience is not enough (data) to safely assign probabilities”

A recent exchange (Casey et al., 2016; Meakin and Jamieson, 2016) raised the latent issue of whether, and to what extent, expert experience forms an acceptable basis for assigning probabilities. As asserted in Champod (2014), and reiterated recently in Meakin and Jamieson (2016), the critical issue is disclosure of data and making it available early enough in the process in order to allow for a proper consideration by the defense. The deeper issue, however, appears to lie in the notions of expert experience and so-called “personal” probability assignments. The ENFSI Guideline, for example, mentions expert experience as one possible source for informing the process of probability assignment: “Such data can take, for example, the structured form of scientific publications, databases or internal reports or, in addition to or in the absence of the above, be part of the expert knowledge built upon experiments conducted under controlled conditions (including case-specific experiments), training and experience” (Willis et al., 2015, p. 19). This should not be read as meaning that a vague reference to personal experience is on a par with other, more structured data. This would amount to misconceiving the fact that there is actually a hierarchy in the data, with a clear preference given to scientific publications and otherwise widely accessible scrutinized data.

#### 2.2.3. “Evaluations given activity level propositions are massively vague and hence cannot be trusted”

This objection may be the result of the discomfort that can be experienced when faced with incomplete knowledge about factors that influence the assessment of the probative value of scientific observations. However, incomplete knowledge, and hence uncertainty, do not *per se* prevent the conduct of science and its operational use in legal proceedings. What is more, in all parts of legal and everyday practice, one needs—inevitably—to act *despite* knowledge being incomplete. It is the very task of science, thus, to quantify the extent of available knowledge so that it can be used in an informed way. The reply “it's possible”^11^ when confronted with the event of transfer or contamination, as scientists still often do in criminal proceedings, is a vague statement and is not a quantification of knowledge as we understand it in the discussion here.

The view that partial knowledge can be used is challenged, for example, when outcomes are subject to variation and scientists refrain from addressing them, equating variation with “no knowledge” about the topic. Forensic examination of glass provides a telling illustration for this. Research has shown that the quantities of glass fragments transferred to the surfaces of the clothing of the breaker vary considerably even for experiments with the “same” controlled conditions (e.g., regarding mode of breaking, distance between the breaker and the window, etc.). But does this mean that we “know nothing”? Clearly, scientists *have* knowledge about the phenomenon of glass transfer^12^ in the sense that they won't expect to encounter all possible numbers of fragments with the same probability. For example, depending on factors such as the distance between the breaker and the window, the mode of breaking, the time since window-breaking and seizing a suspect's clothing etc., scientists may consider it more probable to recover less than, say, five glass fragments, rather than more than five. It is the scientists' core task to elicit and convey such expressions of expert knowledge, because no one else in the proceedings is in a better position to do this. It may be a challenge for scientists to provide probabilities for recovering exactly 0, 1, 2, … fragments (although simulation approaches exist e.g., Curran et al., 1998), but it is feasible also to choose a strategy going from the general to the particular, starting with probability assignments for apportionments of fragments such as “none,” “few,” “some,” and “many.” This helps break down the difficulty of probability assignment and make particular assignments more intersubjectively acceptable.

What is important is not the variation *per se* but how different the expected outcomes are given both propositions. Imagine that 2 min after a window is broken, a person is arrested and his sweater searched for glass. More than 80 fragments (sharing the same physical properties as the broken window) are recovered. Is this result more probable given that he broke the window or given that he had nothing to do with breaking incidents? In some breaking experiments, it was observed that the number of fragments transferred was between 44 and 241 (with a mean of 127, see Hicks et al., 1996). So, there is variation, but when one looks at persons who have not broken windows and searches their garments, one finds that, in general, on sweaters, there are only between 0 and 2 fragments (sharing the same characteristics, see Coulson et al., 2001). Thus, clearly, finding 80 fragments is much more probable given one proposition than the other, *despite* the variation observed. Using the data from both surveys, one can assign a probability to the results given each proposition. In cases involving DNA, most studies focus on one activity only, but what is important is the comparison of the probability of the outcomes given both the alleged activity and at least one alternative. This comparison will then enable us to see if the variation that we have observed has an impact or not on our conclusions.

One further point that warrants a comment is “vagueness.” We would strongly advise against the use of this term as a qualifier for a forensic evaluation *if* that evaluation has been conducted thoroughly. Let us recall again the undisputed starting point: there is variation in findings. What scientists do is to accommodate this *through probability*. What does this mean? It means that scientists will assign probabilities to the various outcomes depending on the extent to which they expect them to occur. For example, consider a case in which a victim has been punched several times to the head, resulting in profuse bleeding. Given the proposition that the suspect (arrested immediately after the assault) is the assailant, we can postulate one main, potential outcome: finding, on the suspect's fist, blood with a DNA profile corresponding to that of the victim. A fairly high probability (i.e., toward the upper end of the range between 0 and 1) may be assigned for this particular finding. By coherence, other findings such as no blood at all, or blood with a profile different from that of the victim will thus be assigned lower probabilities (i.e., toward the lower end of the range between 0 and 1). So, there is variation in the potential findings but there is a major finding that dominates our expectations. Consider now a different version of this case, in which the assault was less violent, did not result in the bleeding of any protagonist, but involved several victims. In such a case, it may be necessary to specify a broader range of potential findings, possibly including mixtures. In the event that the person of interest is the assailant, there may not be one outcome that stands out over all the others. Instead, one's probabilities for several of these potential outcomes may be quite similar. Thus, probability will be distributed over several outcomes, with no outcome receiving a probability assignment close 1. The assigned probabilities will *express less strong beliefs* (in the various outcomes) but—and this is the important point—*this does not mean that the assignment as such is vague*. Less strong beliefs simply reflect the fact that one is less affirmative. Every statement of probability expresses a particular state of uncertainty, that is a well defined opinion, but none of these expressions are deficient if they are derived properly to reflect the expert's current state of knowledge.

So, even if the scientist may report a neutral finding^13^, due to limited expert knowledge (to enable the assignment of probabilities that are different in the numerator and the denominator), this is still an important evaluation to present to the fact-finder. If nothing else, it will inform the decision-maker that they need rely on non-DNA evidence to decide the case.

### 2.3. Reporting

#### 2.3.1. “It is impossible to know from the quantity of DNA obtained, and the quality of the profile, whether the DNA was deposited by direct contact or indirect transfer.”

The concern expressed in the section title is also sometimes seen as the problem of whether we can determine or, as noted by some discussants, “deduce” whether a given finding is the result of primary or secondary transfer. The misconception here is not to understand that the process is not deductive^14^, but remains inductive. Hence one cannot “know for sure”—but one can offer guidance, in the form of probabilities for the results, to help fact-finders decide on the truth of the propositions of interest.

More generally, the claim that particular observations do not allow one to draw categorical conclusions about a particular activity is uncontested and also holds for many, if not all, types of forensic traces. Taking glass as an example, no proficient forensic scientist would conclude that finding a number *x* fragments is the result (or the probable result) of smashing a given window. Similarly, finding a number *y* particles of gunshot residue does not allow one to say that the person of interest discharged a firearm, to the exclusion of other propositions. The impossibility of such direct “jumps” from observations to conclusions in these examples does not derive, however, from the fact that the trace material is present in small quantities. The shortcoming in the reasoning also holds for the so-called macro-traces. To illustrate this point, imagine that large quantities of fresh blood are observed on the hands of a person of interest. Such a result does not entitle one to argue that the exclusive or probable cause is stabbing the victim. Depending on the case circumstances, trying to help the victim may also be a viable proposition.

As discussed, the scientists' task, when operating in evaluative mode, is not to “infer activities” but to provide expressions of probative strength to help the court discriminate between competing propositions regarding activities. This requires the scientist to assign probabilities for the DNA results as obtained in the case at hand given each of the propositions of interest. The fundamental question associated with probative value then is: “Under which of the competing propositions regarding activities do we consider the findings more probable?” It may be that scientists think that they have no reason to consider the observations more probable in one version of the events than another. But this will not be a defect of reporting given activity level propositions, nor of the framework of evaluation. It only means that, in the current state of knowledge, the findings do not have any discriminative capacity (in a technical sense, such results would have a likelihood ratio of 1). As discussed, this is a well-defined result and should be reported, so that people are not prosecuted on the basis of forensic results that are not probative at this stage. As much as it is useful for a recipient of expert information to hear when observations support one proposition rather than another, and (if possible) to what extent, it is useful for them to know when findings do *not* allow them to alter their beliefs in the propositions of interest.

In other terms, it is not a matter for the scientist to say whether a proposition, such as “Mr. A stabbed the victim (i.e., the DNA is from primary transfer),” is true, given the forensic observations, but the extent to which she expects to see these observations, given the proposition “Mr. A stabbed the victim.” The scientist should be assessing the probability that DNA would be transferred, that it would persist and that a matching profile would be obtained, given the truth of this proposition. But there is one more dimension to the latter question. In order to be balanced, scientists must not only think about their results, given one activity, but also given at least one alternative activity (for example, that the suspect handled the knife innocently after the incident), and assess whether, and if so to what extent, the observations are more probable given one activity rather than another. It is therefore of paramount importance that scientists do not confuse the probability of primary transfer with the probability of observing the results if Mr. A stabbed the victim. We agree that the difference is very subtle and this is a reason why, in the propositions, one ought to describe activities and not use the terms “primary/secondary transfer^15^.” This allows one to distinguish, on the one hand, what the court will assess (i.e., activities), and on the other hand, what the scientist will assess, that is the probability of observing the results given the activities. One of the terms used to assign the latter probability is commonly known in the literature as the “transfer probability” (Evett, 1984; Evett and Buckleton, 1989)^16^. We thus stress that a transfer probability focuses on the findings, given propositions, *not* the reverse.

#### 2.3.2. “You cannot say that he stabbed the victim! the only thing that DNA allows you to say is that he had recent direct contact”

This objection is often heard from recipients of expert information when the propositions of interest in the scientist's report are formulated closely to the specific actions that define the crime. For example, propositions such as “he handled the knife,” “he punched the victim,” “he fired the gun,” may provoke such objections. It is often felt that less specific formulations such as “he is in contact with” are more appropriate. However, this objection stems from a misconception about the role of the scientist with respect to the propositions. As noted at the end of the previous Section, by writing down propositions in their report, scientists are only “setting the context” in which the findings will be assessed. That context is given to the scientists by the court and/or the parties and this context naturally relates to the alleged actions. Hence, scientists do not express any opinion directly on those propositions, notably regarding their truthfulness, adequacy or otherwise. The scientist's reporting only focuses on the weight to be assigned to the DNA findings in light of these propositions. Scientists should not suggest in any way that they are in a position to say, for example, that “he handled the knife,” or that “he was in direct contact with the knife.” If they do, they fall for the same fallacious thinking explained above. The only opinion they are allowed to express is in relation to the probability of the DNA findings if one or the other proposition is true. Specifically, when the scientist writes that “this amount of DNA is what we expect if Mr. A. stabbed the victim,” the scientist is reporting only about the DNA results, and is not taking any stance on whether or not Mr. A. stabbed the victim. The latter is simply what is alleged by the parties in their own terms.

#### 2.3.3. “Because many lawyers may lack awareness as to the problem of transfers, analysts should flag the issue in their reports whenever the analysis process suggests that various transfer mechanisms may explain the findings”

Explaining the observations is a procedure that would be acceptable for the scientist to perform if they were at the investigative phase and not being asked to evaluate the forensic biological results in the context of the case, at court. To clarify this point, it is useful to recall the following two fundamentally different perspectives. In the investigative phase, observations are taken as a starting point. They are used to suggest what happened (i.e., activities). For example, one takes the finding of small quantities of DNA on the suspect's shoe as a starting point to suggest that the suspect was the person who kicked the victim. The other perspective takes propositions as a given (as it would be the case in court), to reason about the findings. One assumes that the suspect is the person who kicked the victim, and then one reasons about the kind of trace pattern one would expect to observe on the suspect's shoes. In evaluation, it is the latter perspective that is appropriate for forensic scientists. As noted by Margot, “[w]hether these results could be observed if one proposition for the event is true rather than another proposition is the central relevant matter on which the forensic scientist may comment” (Margot, 2011, p. 796). Note however that there may also be more than two propositions of interest.

At this juncture we would like to include a brief note on the distinction between explaining the observations^17^ and evaluating them, as well as the difference between explanations and propositions (Evett et al., 2000a). We often hear that, after scientists do all the complex evaluations that activity level propositions may require, and provide their results on the stand, the defense are just going to suggest an explanation. For example, the defendant may argue that he spat on his hand as he was walking down the street and touched a bench on which the victim later sat, or some other explanation. It is worth stating that this is explaining the results, and that the defense^18^ provides such explanations once the results are known. Therefore, such explanations are generally based on the results and may not be based on the relevant circumstantial information in the case. Such explanations do not count as acceptable, formal propositions, because one cannot meaningfully assess the probability of the results given explanations that themselves are merged with the results (i.e., this would constitute circular thinking). Explanations are generated *post-hoc* in order to account for the results. They can be statements of the blindingly obvious, they can be speculative or they can be fanciful, having no logical connection with the circumstances of the case, even to the point of having no grounding in reality (see in particular Evett et al., 2000a and more recently Jackson et al., 2013 for examples and detailed discussion). In contrast, propositions are formal statements of competing allegations or suggestions that are dictated by the relevant circumstances of the case and not by the results themselves. So if changes are suddenly brought up at trial, scientists need to be careful not to give *ad-hoc* assessments where evaluation would require detailed checks with relevant literature and specialized knowledge. This is also why, for example, the ENFSI Guideline (Willis et al., 2015) emphasizes that scientists should mention in their report that their evaluation is based on their understanding of the relevant circumstances at the time of writing the report and that if any assumptions or information is incomplete or incorrect, they will have to re-evaluate their findings^19^.

The above distinction between explanations and propositions is crucial and it is worth to summarize and relate it to standard notions from other inferential disciplines. Characteristically, explanations account for—or are made to “fit around”—the findings that have been made in a case. Explanations entail a deductive mode of reasoning as they seek to explain existing results, typically in causal terms. As such, forensic explanations are generated and considered after relevant observations are known. Often, the generating process for explanations results from abductive reasoning not limited to the forensic scientist, but may also extend to case investigators. As such explanations are theoretically open-ended, with no limit on their number though some of the explanations may be more or less fanciful (e.g., not testable in a logical sense), implausible or even speculative than others, to the point that no meaningful probability may be assigned to them. Unlike explanations, propositions are formal statements that can be clearly related to the case context and subjected to a proper inductive mode of reasoning.

We would suggest that consideration and proposal by a scientist of the various possible modes of transfer to account for DNA findings may be of use in the investigative phase of a case. But scientists should not *systematically* explore and comment on all conceivable mechanisms of transfer (so-called caveats) in their statements (but may do so within the lab-file, or in a “Technical Issues” section of the report Evett et al., 2000b). Moreover, when a case enters the evaluative phase, and particularly when in court, a scientist should resist offering a view on explanations for transfer but concentrate on evaluating probabilities for the results given formal propositions based on the circumstances of the case. Advancing explanations at the evaluative stage amounts to treating transfer dismissively, rather than considering its impact on probative value in a formal and explicit way.

#### 2.3.4. “The safest course is for an analyst simply to report the results of the DNA test, alert both counsel and the jury to the possibility of transfer, and leave the jury or factfinder to assess their implications”

This argument is a similar to the previous one, about caveats, but here the burden of how to assess the implications of transfer is left to the factfinder. We are of the opinion that *leaving the factfinder to assess implications of transfer* threatens the appropriate conduct of the forensic findings: if scientists do not—or cannot—evaluate their results, then how could the factfinder do so? Hence we find this position problematic. Clearly, proceeding in this way is an easy course for scientists, because it reduces their task to technical reporting, but it could be very misleading for innocent defendants because findings will be left uninterpreted at the propositional level that really matters (i.e., activity level). Forensic scientists have (or should have) specialized knowledge on transfer and persistence, as shown by publications in forensic journals, and they therefore have the duty to report the value of their results at that level. If the knowledge is not sufficient, then scientists must tell the instructing magistrate or the court (or preferably even before, earlier in the process) that, as a consequence, their results do not help discriminate between the propositions at hand. We do not believe that—in the evaluative phase—scientists should provide a list of all theoretically possible modes of transfer of DNA (see also Section 2.3.3). If the scientist were to provide such a list, how does the court choose which is the most likely mode of transfer? This would leave the court in the difficult position of having to choose which of a potentially large number of possibilities (that are not necessarily exhaustive) is the most likely, without being able to rely on any specialized knowledge to do so. However, we do believe that it is the proper role of the scientist to talk generally about transfer and persistence of DNA (see also Section 2.3.3).

An intricacy related to the above is the use of the term “possible.” As human beings, we refer to a lot of events as being “possible” (i.e., the probability of the event is not 0), but forensic scientists should be more informative than this: they should assess *how probable* their results are given the propositions at hand, just like they do when they assess the probability of observing a given DNA profile if it came from some unknown person. If a scientist were to be asked “what is the probability of obtaining a matching DNA profile if it came from Mr. A or if it came from someone else who happens to have, by coincidence, the same profile,” which is an explanation, the scientist would have to answer that those two probabilities would be the same (i.e., approaching 1). Therein lies the problem for the scientist and the court, generating explanations leads to probabilities for the results of a value approaching 1. Provision of explanations is deeply rooted in general forensic science thinking and we regularly see reports in which the scientist writes “It is possible that this DNA comes from Mr. A. But, it is *also possible* that it comes from his brother or an unknown person.” This sort of explanation-based answer is unsatisfactory because it leads the scientist to opine directly on a proposition (see also Section 2.3.2 regarding the role of the forensic scientist).

#### 2.3.5. “When scientists are unable to evaluate their observations given activity level propositions, then they should retreat to evaluations given source or sub-source propositions”

This claim rejoins Section 2.3.4, which refers to the claim that an evaluation given source level propositions is a “safe course” for forensic scientists, when they cannot help address activity level propositions. However, what a safe course of proceeding is for the scientist may again not be so for other participants in the process^20^. The problem is that the “safe course” for the scientist inevitably restricts the evaluation to the (sub-) source level. Consequently, the court is given no guidance about how to evaluate with respect to activity level propositions. If the court confuses the scientist's (sub-) source evaluation with the activity level evaluation, then there is a risk that this may lead to a miscarriage of justice^21^.

For the above reason, recent recommendations by ENFSI specify that the choice of source level propositions is limited to well defined situations, that is “(…) cases where there is no risk that the court will misinterpret [the findings] (…) in the context of the alleged activities in the case” (Willis et al., 2015, p. 12)^22^. But for small quantities of trace material, this is rarely if ever the case, because such traces require expert knowledge “(…) to consider factors such as transfer mechanisms, persistence and background levels of the material which could have an impact on the understanding of scientific findings relative to the alleged activities” (Willis et al., 2015, p. 11). For all of these reasons, the ENFSI guideline concludes that “(…) the choice between (sub-) source and activity should not be influenced by the availability of data or expert knowledge but solely from the consideration of factors such as transfer, persistence and background levels that could crucially affect the strength of the findings within the context of the case circumstances” (Willis et al., 2015, p. 13). This includes a statement of limitations as to the data and the individual expert knowledge (see also Section 2.2.1).

An objection that may be raised against the position outlined above is its feasibility. That is, although activity level propositions may be recognized as the relevant propositional level, specialized knowledge necessary for evaluation given this level of propositions may be unavailable. A natural consequence of this starting point would be not to introduce the results at trial, in order to protect defendants against unwarranted interpretations in such cases. However, there appears to be no consensus among scientists on how to proceed in such situations. Some scientists maintain their intention to report findings given source level propositions *although* they are clearly unable to help with the issue of activities. As a consequence, we do not subscribe to this view *of retreating to evaluation given (sub)source level propositions*, and neither does the ENFSI Guideline, which requires scientists to clearly acknowledge that their evaluation falls short of the real needs. In Guidance note 2, the Guideline states that “(…) if the examiner chooses (…) to report the findings at source level (…), the examiner shall explicitly state that the rarity of the profile does not address the question of the relevance of the findings in relation to the alleged activity” (Willis et al., 2015, p. 14).

Proponents of the view according to which uninterpretable results should be mentioned at trial appear to misconceive the fact that different stages in the forensic process have different requirements (Jackson et al., 2006, 2013). It is beyond dispute that, at an investigative stage, scientists can help the process move on when they factually report about the observation that a defendant's traits are also observed in trace material (e.g., in the case of mixtures). This is useful information for selecting possible candidates on whom to focus further investigations. At trial, however, the requirements are different. At trial, the defendant has already been selected, and if DNA is to play any further role, it must be given a weight (Evett, 2015)—not against any propositions, but propositions at the relevant level.

#### 2.3.6. “When evaluating forensic DNA traces given activity level propositions, the scientist infringes on the duties of the court”

A perception encountered among legal practitioners, as noted earlier in Section 2.3.2, is that when evaluating DNA traces given postulated activities, scientists take on the role of the fact-finder. This observation is a cause of concern because it does not reflect the scientist's intention and laying bare this misconception is challenging. We think that there is merit in reiterating that it is not for the scientist to give an opinion on whether the transfer is primary or secondary (or the probability that the transfer is primary or secondary) because giving such an opinion would amount to giving an opinion on the propositions of interest, for example whether “Mr. A had sex with Ms. A” (transfer was primary) or “Mr. A and Ms. A attended the same party, but had no particular interaction” (transfer was secondary). Clearly, this is a question for the court.

The above distinction is very subtle, for all discussants, including scientists. It comes down, in one way or another, to the problem of the transposed conditional. Many authors have formally described the contribution of the scientist and the nature of expert opinion in the criminal justice system, with the one key aspect being that the scientists' role is to evaluate their results given the competing propositions regarding activities, and that it is for the court itself to assess the truth of the propositions. Unless scientists are operating at the investigative stage, they should express probabilities only for their results given propositions, but not the reverse. An example of a relevant statement would be: “The probability of observing this quantity of DNA if Mr. A had sex with Ms. A as alleged by the prosecution is in the order of 0.6, whereas the probability of observing this quantity of DNA if Mr. A had social interactions as alleged by the defense is in the order of 0.01. This means that it is about 60 times more probable to observe this DNA result if the prosecution's case is true rather than if the case of the defense is true.” However, there is the risk that the receiver of this information will interpret the low probability for the findings, given the alternative proposition, as meaning there is a low probability for the proposition, given the findings—a reasoning error that is known as “transposing the conditional,” and which was not intended by the scientist. This is why some reporting agencies explicitly mention, in their written reports, examples of sentences of what their conclusions do *not* mean.

## 3. Conclusions

From the discussion presented throughout Section 2, three main points emerge:

First and foremost, forensic interpretation, as conducted by the scientist, focuses on the observations, not on the propositions. Stated otherwise, the question for the scientist is “What is the strength of these findings with regards to the propositions of interest?” The scientist should *not* attempt to answer the question “How probable are the propositions *given* the findings?” Scientists do not evaluate and provide an assessment of the probative strength of scientific findings when they express opinions on propositions. Hence—for the scientist—evaluation given activity level propositions does *not* mean to opine, probabilistically, on competing activities that may have “caused” the findings. Evaluating forensic results means to provide information that helps the recipient of expert information discriminate between propositions, whatever their belief in those propositions is prior to hearing the scientific findings.
Second, reporting on the probability of the observations given competing versions of the case, regarding activities, does not exclusively depend on numerical data, but is also informed by expert knowledge and experience, for which scientists can provide appropriate documentation and demonstrate how it shapes their opinion. What is more, the scientist invokes information that is available for disclosure and auditing. An important corollary of this is that even though task specific data may be unavailable or scarce, it does not mean that no probability can be assigned. In particular, this is not to suggest that any opinion, or mere guesswork, is a valid substitute for thorough scientific assessment. It highlights the need for the elicitation of expert probabilities and knowledge through formal methods and techniques, known also in other areas of specialization, such as risk and safety assessments (e.g., O'Hagan et al., 2006; Aven and Reniers, 2013). There is merit in further developing these approaches for forensic science applications, as well as strengthening the body of structured knowledge (i.e., relevant data on phenomena such as transfer and persistence) for various types of forensic traces (Evett, 2015). This rejoins the idea of developing a knowledge base system that would include experiments and exemplar probabilistic models for evaluation (e.g., BNs). This is widely considered a critical step that the field needs to take now.
Third, variability in the observations (e.g., with respect to quality and quantity of transferred material) observed in experiments under controlled conditions, is both natural and expected. It does not mean that such data cannot be used for evaluation in actual cases, nor does it mean that no conclusion may be drawn. This view is also supported by professionally organized forensic caseworkers (Casey et al., 2016). Variability is an inevitable feature of scientific experiments, observations and measurements, and produces uncertainty. The scientific approach to such uncertainty is to capture it by probability and to take it into account in the scientist's evaluation (e.g., it will be ensured that the data used come from experiments that relate directly to the analytical methods used in the case of interest). Therefore, variation *per se* is not a primary matter of concern; what does matter for the scientist is to see whether the probability of the outcomes given different propositions varies. That is, for the results to be useful, the outcomes need to be more probable given one version of the case (i.e., proposition) than given an alternative version of the case. It is on this latter issue that the scientists need to focus their attention.

The above observations diffuse the call for so-called “unpredictable” forensic DNA traces, in particular low quantities, to be withheld from being used in the process. This is so because the perceived drawbacks, although inspired by known difficulties, do not properly acknowledge additional levels of scientific observations (e.g., extrinsic features such as the quality and the quantity of recovered material, and the position in which it was found) that may be available and that characterize a comprehensive evaluation of forensic results. This perspective goes beyond the mere assessment of the rarity of the analytical features (i.e., the genetic profile). Indeed, for decades, forensic scientists and recipients of expert information have found comfort in seeing forensic DNA analyses provide “constant” and “stable” results in the sense that the DNA profile observed for a sample from a person of interest will, broadly speaking, be observed to be the same for a stain left by that person - as long as quality and quantity of the staining are appropriate, and the chain of custody is impeccable. To a large extent, this has led to technical efforts and investments being spent on ensuring that analyses will reveal the same profile for materials that come from the same source. This is, undoubtedly, an important preliminary requirement for use in forensic science. Unfortunately, however, this perspective was accompanied by the idea that all that is necessary to assess the strength of the findings is an assignment for the probability of observing the profile of interest for an unknown person. This focus on analytical accuracy and rarity of features conflicts with the intricacy of additional dimensions that DNA profiling entails, such as the very fact of finding DNA at a particular place on a receptor surface (i.e., extrinsic aspects). Stated otherwise, what we have come to see now are conventional interpretation schemes conditioned mainly on source (or sub-source, e.g., in the UK) level propositions being applied to questions, issues and challenges for which these schemes have not been designed, and this has the potential to create stupefying situations in which reports on forensic DNA results are at odds with the case as a whole^23^. Worse still, evaluation given sub-source and source propositions alone can lead to an over-valuing of the scientific evidence, risking miscarriages of justice (Gill, 2014; Jackson, 2014).

The above calls for a readjustment of perspective. To ensure that forensic DNA results are meaningfully used in the legal process, scientists must work on improving their knowledge and understanding about additional factors that characterize not only intrinsic features (e.g., DNA profile) but also extrinsic features (e.g., location where DNA was found). This call is not new (e.g., Evett and Weir, 1998; Taroni et al., 2013; Champod, 2014), but we see that the field is rather reluctant and awareness increases only slowly. At the same time, reports accumulate on real cases (e.g., Gill, 2016) where DNA turned out to be a source of conflict essentially because the key issues for the court related to activities whereas the scientist evaluated the findings in light of questions of source. Thus, evaluation given activity level propositions corresponds to a real need and we foresee that both prosecution and defense counsels will intensify their probing of forensic science regarding this propositional level, not least because recently issued guidelines (i.e., Willis et al., 2015) on evaluation and reporting explicitly set this forth as the standard of interpretation. Achieving this standard is a delicate and challenging endeavor because it operates at the frontiers of current knowledge. However, by gathering, sharing and organizing specialized knowledge in a structured and systematic way (i.e., a shared knowledge base), the forensic community as a whole has the potential to work toward (i) increasing the number of cases in which findings can be assessed given activity level propositions, and (ii) rendering activity level evaluations more trustworthy in those cases where such evaluations are feasible.

In this paper, a discussion format has intentionally been chosen. The aim was to concentrate and restate replies to recurrent objections to emphasize on the need to pursue this topic from a broad perspective, associating both forensic scientists and lawyers. In view of all the arguments presented, our view is that evaluation given activity level propositions represents a main point of the agenda of future research. Besides the justified calls for more structured expert knowledge, we also recognize the need to report on more practical case examples that demonstrate the feasibility of this perspective in a way that practitioners can understand. Such reports on practical examples exceed the space available in this communication, but is the object of ongoing collaborative work between the authors.

## Author contributions

All authors listed, have made substantial, direct and intellectual contribution to the work, and approved it for publication.

### Conflict of interest statement

The authors declare that the research was conducted in the absence of any commercial or financial relationships that could be construed as a potential conflict of interest.

## Footnotes

^1^DNA is chosen here because it is widely practiced. It goes without saying, however, that our discussion is equally applicable to other types of transfer trace materials, such as glass fragments, fibers or gunshot residues (GSR).

^2^Note that sub-source level propositions (Evett et al., 2002) are defined by replacing the bodily tissue “trace” (e.g., bloodstain) in the proposition by “DNA.” For the remainder of this paper, we will focus on activity level propositions so that the distinction between source and sub-source levels is irrelevant. See also Cook et al. (1998) on the concept of hierarchy of propositions.

^3^It is worth indicating here that case circumstances are as important as propositions and that one will need indications such as alleged activities and timing.

^4^By all results we mean not only the DNA profile, but also aspects such as the quality and quantity of staining, and the position where it was found.

^5^Throughout this paper we will, at times, refer to the expression “evaluation in court” even though we intend our arguments to apply to the evaluative stage at large which, in some judicial systems, does not necessarily take place in court.

^6^The titles throughout Section 2 reflect our perceptions and summaries of recurrently encountered concerns. Specific references are cited within each subsection.

^7^In all criminal justice systems that we know of, it is the right of defendants to remain silent and not incriminate themselves. Also, it is often considered strategic for the defense to make a statement as to the evidence only after the results of the analyses are known.

^8^It is generally understood that scientists should concentrate on information that is relevant for the task at hand (i.e., so-called task-relevant information), keeping in mind challenges posed by human factors.

^9^On this point, see also Casey et al. (2016), who argue “(…) not evaluating DNA evidence in case work is potentially more dangerous and reckless than carrying out an evaluation based on limited datasets.” We concede that this topic is delicate and it is important not to suggest that scientists allow themselves to suggest an answer as they please and hide behind statements such as “I am an expert.”

^10^While there is substantial literature available on BNs for evaluating forensic DNA results (e.g., Biedermann and Taroni, 2012), BNs remain inaccessible to many biologists, mainly because of lack of training. An easily accessible repository with freely available examples that can be utilized with open-source software would be an asset to complement the idea of a knowledge base, as mentioned above.

^11^The same applies to “could have”; and there have been several notable judgments where courts have ruled against the unqualified use of such phrases.

^12^A further relevant factor is background presence. Regarding DNA, there is limited knowledge about naturally present DNA in the environment, which is especially important in cases where the defendant and victim cohabit (as in the Amanda Knox case, for example Gill, 2016).

^13^In more technical language, this would correspond to a likelihood ratio of 1.

^14^On the notion of deductive logic, see also Jackson et al. (2013), for example.

^15^For more on this topic, we refer the reader to Hicks et al. (2015).

^16^Note that there are also phenomena of persistence and detection/recovery to be taken into account.

^17^While technically the word “observations” is to be preferred, we will be using the more colloquial word “results” to refer to the outcome of the scientist's analyses.

^18^The problem of *post-hoc* rationalizations is not restricted to the defense as explanations can also be brought up by the prosecution. See, for example, the bleach cleaning hypothesis in the Amanda Knox case (Gill, 2016).

^19^Interruptions in the proceedings can be granted by the court both in inquisitorial and adversarial proceedings, and will usually be granted if the question is of importance to the court.

^20^See for example cases such as Jama (Gill, 2014, p. 27) or the Ruelas Case (Murphy, 2015, p. 56) which illustrate that the sole consideration of sub-source issues does not accommodate and represent an evaluation of the joint probative value of the results from different swabs, the quantities of DNA found, nor the presence or absence of other trace materials. When considering activity level propositions, all observations should be assessed, which is what is needed.

^21^See http://www.scientificamerican.com/article/when-dna-implicates-the-innocent/ for recent case involving a stupefying situation in which a forensic DNA report found a correspondence between the DNA profile of a hospitalized person and DNA found on a murder scene. It appeared that the same paramedics treated the hospitalized person but also worked on the crime scene a few hours later (see also https://californiainnocenceproject.org/2013/06/how-an-innocent-mans-dna-was-found-at-a-crime-scene/).

^22^This may be the case, for example, when there is a large and fresh bloodstain at the point of entry (e.g., broken window) and it is not contested that the blood stain is relevant to the case (i.e., the trace is a direct consequence of committing the burglary).

^23^See also the case mentioned in footnote 21.
